# Brain-Machine Interfaces to Assist the Blind

**DOI:** 10.3389/fnhum.2021.638887

**Published:** 2021-02-09

**Authors:** Maurice Ptito, Maxime Bleau, Ismaël Djerourou, Samuel Paré, Fabien C. Schneider, Daniel-Robert Chebat

**Affiliations:** ^1^École d’Optométrie, Université de Montréal, Montréal, QC, Canada; ^2^Department of Nuclear Medicine, University of Southern Denmark, Odense, Denmark; ^3^Department of Neuroscience, University of Copenhagen, Copenhagen, Denmark; ^4^TAPE EA7423 University of Lyon-Saint Etienne, Saint Etienne, France; ^5^Neuroradiology Unit, University Hospital of Saint-Etienne, Saint-Etienne, France; ^6^Visual and Cognitive Neuroscience Laboratory (VCN Lab), Department of Psychology, Faculty of Social Sciences and Humanities, Ariel University, Ariel, Israël; ^7^Navigation and Accessibility Research Center of Ariel University (NARCA), Ariel, Israël

**Keywords:** blindness, cross-modal plasticity, sensory substitution device, visual prostheses, sensory substitution

## Abstract

The loss or absence of vision is probably one of the most incapacitating events that can befall a human being. The importance of vision for humans is also reflected in brain anatomy as approximately one third of the human brain is devoted to vision. It is therefore unsurprising that throughout history many attempts have been undertaken to develop devices aiming at substituting for a missing visual capacity. In this review, we present two concepts that have been prevalent over the last two decades. The first concept is sensory substitution, which refers to the use of another sensory modality to perform a task that is normally primarily sub-served by the lost sense. The second concept is cross-modal plasticity, which occurs when loss of input in one sensory modality leads to reorganization in brain representation of other sensory modalities. Both phenomena are training-dependent. We also briefly describe the history of blindness from ancient times to modernity, and then proceed to address the *means* that have been used to help blind individuals, with an emphasis on modern technologies, invasive (various type of surgical implants) and non-invasive devices. With the advent of brain imaging, it has become possible to peer into the neural substrates of sensory substitution and highlight the magnitude of the plastic processes that lead to a rewired brain. Finally, we will address the important question of the value and practicality of the available technologies and future directions.

## History of Blindness

For most sighted people, the very thought of blindness awakens a deep fear: a fear of the unknown, of an “endless night,” of being unable to move and orient oneself (Commend, 2001). This fear has had repercussions throughout recorded history and on the conditions of people living with blindness.

### A Limiting Vision of Blindness: From Ancient World to Enlightenment

Throughout the ages, blindness has long been associated with mythical or biblical beliefs to provide lessons or even to give inspiration to the “common people.” In Ancient Greece, blindness was generally viewed as a punishment from the Gods. Indeed, although Homer was rumored to be blind, the scarce reports that remain of this period depict blindness as being associated with accidents, war injuries and, importantly, punishment for transgressions (Barasch, 2001). That preconception persisted through the Middle Ages when blindness and other disabilities were often viewed as acts of god and deliberate blinding was the most dreaded of punishments (Wheatley, 2010). People living with blindness were thus associated with misery and were often depicted as beggars or as praying for a miracle of the sort attributed to Jesus (Weygand, 2009). Because of this prevailing attitude toward blindness, blind people long found themselves objects of derision or charity, whose existence was often reduced to their reliance on the help of others for daily living (Barasch, 2001; Weygand, 2009; Wheatley, 2010; Margo et al., 2013). This view role, however, began to change and improve in Europe during the Enlightenment of the 17th and 18th centuries. The separation between blindness and biblical beliefs found first expression in William Molyneux’s question addressed in 1688 to John Locke, cited in *An Essay Concerning Humane Understanding*:

*“A Man, being born blind and having a Globe and a Cube […], Let us suppose his Sight Restored to Him; Whether he Could, by his Sight, and before he touch them, know which is the Globe and which is the Cube?”* [from Ferretti and Glenney (2020)].

The question was later entertained by other early modern philosophers such as Gottfried Leibniz, George Berkeley, Adam Smith, and many others. While this purely philosophical question did not directly address the inclusion or education of the blind, it allowed further conjectures about perceptual learning, multi-sensory integration and the capacity of the blind to learn without the use of vision (Ferretti and Glenney, 2020).

### Education Through Touch: From Diderot to Braille and Howe

**FIGURE 1:**
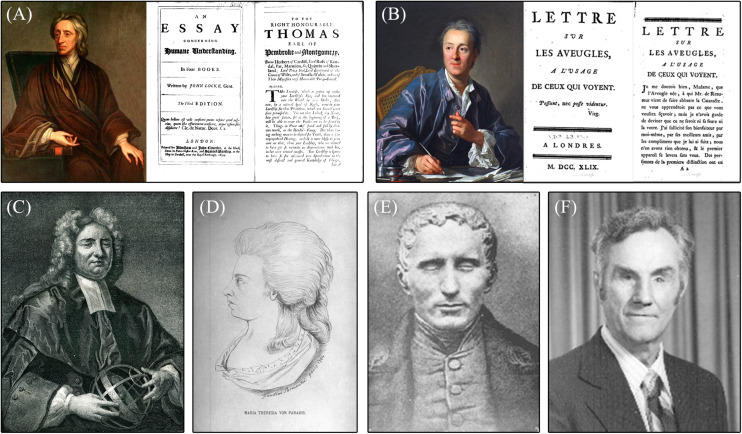
Important individuals in the history of blindness. **(A)** Portrait of John Locke and cover and first page of his paper: *An Essay on Humane Understanding*; **(B)** Portrait of Denis Diderot and cover and first page of his article: *A Letter on the Blind for the Use of Those Who See*; **(C)** Portrait of Nicholas Saunderson; **(D)** Drawing of Maria Theresia Von Paradis; **(E)** Portrait of Louis Braille; and **(F)** Picture of Russell Williams (photo courtesy AER O&M Division Warren Bledsoe Archives, American Printing House for the Blind).

**FIGURE 2:**
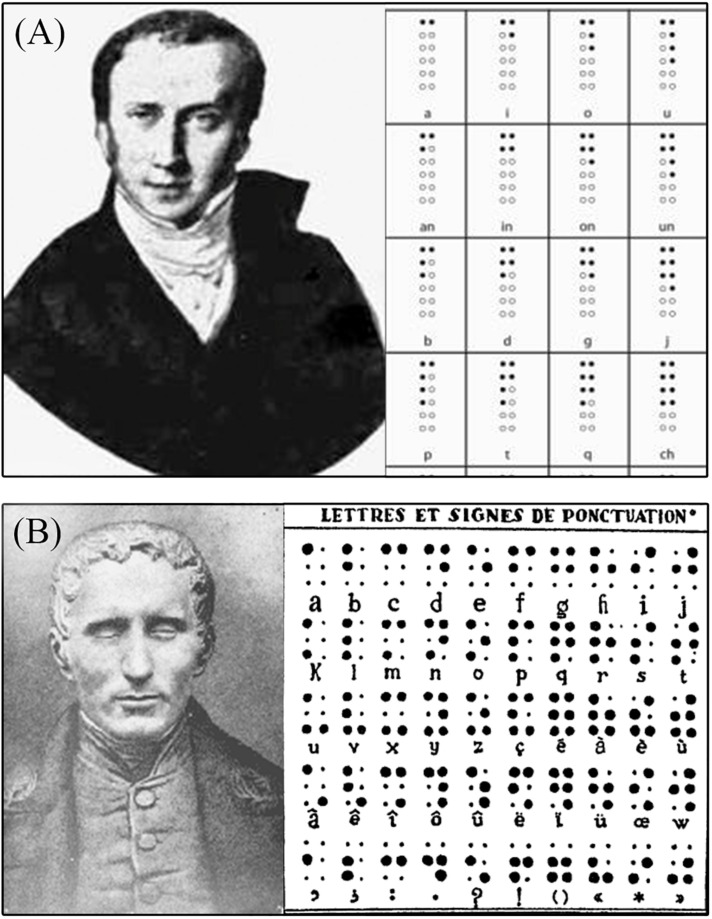
The creation of Braille. **(A)** Portrait of Charles Barbier and examples of his Night Writing system based on phonetics, which is the system that inspired Louis Braille; **(B)** Portrait of Louis Braille and the early version of the Braille writing system.

As education and writing assumed greater importance during the Enlightenment, there arose many examples of blind individuals who successfully educated themselves and accomplished inspirational feats. Notably among them, Nicholas Saunderson (1682–1739), a scholar at the University of Cambridge, became a tutor in mathematics and physics and won the esteem of Newton himself who judged him one of his few contemporaries who truly understood the value of his work. There also were Mélanie de Salignac (1744–1766), a musician who learnt by herself how to read, write and correspond with friends using cutout letters, and Maria Theresia von Paradis (1759–1824), who was a talented singer and pianist (see Figure 1). Such individuals became sources of inspiration for Denis Diderot (1713–1784) in the writing of his 1749 essay *The Letter on the Blind for the Benefit of Those Who See*, where he lauded the abilities of blind people. According to Diderot, educating the blind in writing and reading was possible through the sense of touch (Margo et al., 2013). His philosophy offered a foundation for the efforts toward the education of blind people in the centuries that followed, being one of the first savants who truly focused on their ability rather than disability. Indeed, Diderot’s philosophy was central to Valentin Haüy’s work in founding the first school for the blind in 1784 (now known as the *Institut national des jeunes aveugles* or INJA). Valentin Haüy (1745–1822) was a French calligraphy professor who proved that blind individuals could learn to read embossed text with the use of their fingers. He invented the first reading system of raised Roman letters which he successfully taught for years. Haüy’s school later gave birth to the Braille alphabet, a new tactile writing system invented by one of its blind students: Louis Braille [reviewed in Jiménez et al. (2009)]. Louis Braille (1809–1852) was inspired by the Night Writing (from French: *écriture nocturne*) system previously developed by Charles Barbier de la Serre for the use of French soldiers who had to read and write in the dark while on campaign. Barbier’s system was based on phonetics and consisted of different combinations of raised points on a two by six grid of twelve points. This concept was deemed too cumbersome by Braille, who went to create a two by three grid of six points representing the alphabetical system that was simpler and easier to learn. In 1829, then 15-year-old Louis Braille published his first version of the system, which was officially adopted in the school and in France in 1854. The eponymous Braille system was the first successful sensory substitute for reading without vision and it is still in wide use today. In fact, Braille and the capacity to read through touch constituted a colossal step forward for the rehabilitation of blind people in society, a concept that was promoted abroad by 19th century reformers such as Samuel Gridley Howe, who founded the New England Institution for the Education of the Blind (now the Perkins School for the Blind). Figure 2 illustrates the development stages of the embossed letter system. It is now fully appreciated that blind people can be trained to substitute their intact senses for vision, enabling them to become integral, productive and autonomous members of society. Indeed, the blind can even develop supra-normal sensory abilities through the overtraining of other modalities.

### Understanding Echolocation, the “Sixth Sense” of the Blind

Supra-normal abilities of blind people in other sensory modalities such as touch and audition are well known today, but were first reported as soon as 1749 in Diderot’s work cited above. Indeed, Diderot was among the first to report the blind’s use of echolocation or, as he discussed, their ability to perceive objects and estimate their distance *via* sensations manifesting on perceived on the skin of the face. Diderot attributed this phenomenon to the compression of air against the skin upon approaching an object. According to Diderot, the facial nerves and sensory end organs had increased sensitivity in the blind. Thus, for many years it was held that the blind could feel changes in air pressure with their forehead and cheeks (Burklen, 1924), an ability that was named “*perceptio facialis*,” or “facial vision” (Levy, 1872). At the start of the 20th century, authors began to debate the nature of “facial vision” and on whether it was due to the use of reflected sounds (Dresslar, 1893; Heller, 1904; Truschel, 1906; Villey, 1930), air pressure (James, 1890), “ether waves” (Javal, 1905) or even “vestigial Ranvier corpuscles” in the skin of the forehead (Romains, 1924). This debate continued until the period following World War II, and the conducting of the *Cornell Experiments*, a series of notable experiments where numerous authors systematically investigated the nature of “facial vision” [reviewed in Thaler and Goodale (2016)]. It was then discovered that “facial vision” was not based on atmospheric pressure cues felt on the face but rather that this skill was attributable to the use of auditory cues. When the ears of blind participants were plugged, they were no longer able to use “facial vision” (Supa et al., 1944). This generation of authors understood that blind individuals were using a form of echolocation (as in bats and dolphins) to perceive reflected sounds, sound shadows and changes in the sound (i.e., the Doppler effect) in manners unavailable to sighted people (Supa et al., 1944; Worchel and Dallenbach, 1947; Worchel et al., 1950). It was subsequently concluded that these auditory abilities were however, cross-modally experienced as tactile sensations of pressure against the face (Kohler, 1964), at the conclusion of the long-lasting debate on the nature of “facial vision.” The capacity for echolocation was found to be present in 85% of blind individuals and to correlate with the age of blindness onset and its duration (Juurmaa, 1965). Congenitally blind individuals (CB) proved to be more effective in the use of echolocation than their sighted counterparts (Supa et al., 1944; Juurmaa, 1965; Strelow and Brabyn, 1982; Boehm, 1986). However, it was soon established that blindfolded sighted individuals could learn the skill of echolocation as could individuals who acquired their blindness later in life (Worchel and Ammons, 1953). Thus, “facial vision” (properly echolocation) was and is still viewed as an essential skill for the blind to learn to achieve a higher level of independence. Indeed, in today’s orientation and mobility (O&M) training, blind individuals are taught to use echolocation and environmental sounds in conjunction with the white cane and other technology to navigate safely and independently.

### Toward Independent Travel: The White Cane and O&M Training

O&M training, as we know it today, is still a developing field that traces its roots to World War II (Sauerberger, 1996; Bledsoe, 2010). During those years, blind people were taught to use “facial vision” and other orientation strategies (i.e., memorizing lay-outs and landmarks) with instruction from “orientors” in rehabilitation programs. However, this approach was often prioritized over the cane and other tools that could contribute to the perceived stigma of blindness (Bledsoe, 2010). The numerous American soldiers blinded due to the vicissitudes of war were sent to military hospitals such as Valley Forge and Dibble, where they healed from their wounds and learned to navigate with a cane before being transferred to the rehabilitation program in Avon, Ohio. In order to treat the growing numbers of injured and visually impaired, Valley Forge hired Richard E. Hoover and Warren Bledsoe who had previously worked as teachers for blind individuals. Early in their postings at Valley Forge, they quickly concluded that echolocation alone was insufficient to support safe and efficient navigation during which obstacle avoidance was necessary.

*“[…] the first thing they need is to know how to get around. We’ve been working on it, but not enough. […] People say blind people in this country do a good job of getting around. I don’t think they do a good job. I think they do a hell of a poor job.”* - Richard Hoover [from Bledsoe (2010)].

While we know that canes and staffs have been used for millennia, as attested by numerous examples from ancient Greece and biblical texts (Levy, 1872), the internationally recognized white cane was invented in 1921 and was first promoted by the Lions Club International in 1931. However, Hoover promoted the use of a more lightweight cane and developed the foundations of cane techniques as taught today. Indeed, Hoover blindfolded himself and, alongside Bledsoe and other instructors, experimented with new and affective cane techniques. Based on this experience, he established the “touch cane technique” and trained other instructors in its proper use for the benefit of blinded soldiers. One of those soldiers was Russel Williams, who had lost his sight from injuries during the Normandy invasion. Williams later transferred to the program in Avon, Ohio, where he learned echolocation and orientation techniques, and decided on his own accord to merge all his training to achieve greater autonomy. In 1948, he was appointed as the chief of the new rehabilitation program at the Veterans Administration Hospital in Hines, Illinois. In that time, he worked alongside Bledsoe to enroll and train new specialists in the field of “foot travel,” which later became known as O&M training (Sauerberger, 1996; Bledsoe, 2010). While World War II had disastrous repercussions on the world, it enabled the initiation of greatly improved rehabilitation services offered to individuals with vision impairments, which remain in use to this day. Proceeding from the experiments on echolocation to the development of the touch cane technique, the work following the war enabled the growth of O&M training which now plays a pivotal role in the rehabilitation of visually impaired individuals around the world seeking greater autonomy, confidence, and a better quality of life.

### Modern Technologies: Brain Interfaces to Help the Blind “See”

Technologies and tools introduced in O&M training help blind individuals to expand their perception of the environment and thus extend their domains of action. To date, the white cane remains the main compensatory tool utilized by blind individuals worldwide. As an extension of the arm, the white cane provides safety against obstacles by extending the range of detection and provides additional information (auditory and tactile) on the environment such as changes in floor texture and denivelation [reviewed in Guth et al. (2010)]. However, the white cane, even when used in conjunction with echolocation, has a significant limitation; While it detects objects on the ground, the upper body and head remain unprotected and blind individuals are still at risk of dangerous collisions that they cannot anticipate (Suterko, 1967). Consequently, the blind suffer disproportionately more injuries due to collisions to the head, and are likewise vulnerable to the risk of falls (Manduchi and Kurniawan, 2011) which can contribute to the feeling of anxiety about travels and, ultimately, lead to social isolation (Beggs, 1992). Faced with this issue, it is not surprising that many scientists began working on new technologies aiming to enhance the corporal safety of individuals living with blindness, most ambitiously in efforts to restore sight. These efforts employ brain interface technologies that can tap into the visually deprived brain’s potential of adaptation to new stimuli and tasks. These new brain interfaces can expand the perception of the blind beyond the capacities of the white cane and Braille, thus affording more opportunities to learn, travel safely, and participate as independent members of society.

Today, there are many kinds of brain interfaces aiming to help the blind “see,” which we classified into two main categories of devices. The first category consists of *invasive brain interfaces* that require surgical implementation of the device in the visual system, such as retinal and cortical implants, in order to restore sight to those who lost it. The second category consists of *non-invasive brain interfaces*, such as electronic and electromechanical aids, that aim to complement the sensory abilities the blind already possess, along with sensory substitution devices (SSDs), which aim to offer a “visual-like” experience by electronically translating visual information into another modality, such as touch and audition.

## Brain Interfaces for Vision Recovery

### Invasive Brain Interfaces

Researchers have tested many brain sites for electrical stimulation aiming to restore vision with the use of implants. For example, there have been attempts to electrically stimulate the visual cortex and retinal cells of patients with blindness due to retinitis pigmentosa (RP) or age-related macular degeneration (AMD). In addition, other studies have shown that it is possible to evoke sensory perceptions by stimulating the optic nerve (Delbeke et al., 2001) or the lateral geniculate nucleus (Pezaris and Reid, 2007) although these techniques are not widely used because of the many challenges and risks associated with the neurosurgical procedures to access such inner brain structures (Allen and Ayton, 2020).

#### Cortical Visual Prostheses

Restoring vision has been of interest to scientists for several centuries. Charles Le Roy, a French physicist, was interested in curing diseases with electricity. In an attempt to cure a patient of blindness, he developed a metal device that applied to the head of the patient and connected it to a Leyden jar. Surprisingly for the time, the patient reported perceiving flashes of light during the electric shocks (LeRoy, 1755). This was the first recorded demonstration of the electrical excitability of the visual cortex, and was the inspiration of a series of attempts for vision recovery. In the early 20th century, neurosurgeons made use of the research opportunity presented by awake opened skull patients to electrically stimulate their visual cortex, which evoked the experience of retinotopically organized phosphenes. The spatial representation of the visual field in the human primary visual cortex was discovered using these techniques (Holmes, 1918; Löwenstein and Borchardt, 1918). This approach later prompted John C. Button to develop a device aiming to restore vision to blind people by electrical stimulation of the occipital cortex. In a test of the device, a blind patient reported seeing flashes of light and was able to locate and assess the brightness of a light source (Button, 1958). Some years later, Brindley and Lewin (1968) produced a wireless prototype of a cortical visual prosthesis, which consisted of 80 extracranial radio receivers connected to 80 intracranial electrodes inserted inside the calcarine fissure. The prototype did not support reading as the authors had hoped, but did allow simple pattern discrimination. At around the same time, William Dobelle developed a removable visual neuroprosthesis that allowed him to stimulate the visual cortex of patients undergoing brain surgery [reviewed in Lewis et al. (2015)]. These pioneering studies set the stage for the development of more sophisticated instrumentations and new generation of cortical implants. In 2020, several projects are in progress and clinical trials are underway or planned in the coming years (for a review on neurobionics and cortical implants see: Allen and Ayton, 2020; Chen et al., 2020; on retinal implants see: Nowik et al., 2020).

##### CORTIVIS

The aim of the CORTIVIS project is to capture the visual scene using a bioinspired artificial retina designed to emulate aspects of the visual processing that occur in the retina. The CORTIVIS project uses the Utah Electrode Array (UEA), which consists of 100 electrodes of 1.0–1.5 mm in length. It is designed to reach the cortical layer 4c (the target of geniculate innervations) and to limit damage to neurons. Early experiments showed that the electrical stimulation of the implanted electrodes elicited visual perception in monkeys (Normann et al., 2009) and preliminary investigations were carried out in human patients with epilepsy or brain tumors during brain surgery. Promising results were obtained with safe implantation, high-quality visual cortex recordings and induced perception of phosphenes (Fernandez et al., 2015). Recently, a new system coined “The High-Channel-Count Neuroprosthesis” has been successfully tested on monkeys. It uses a high number of implanted electrodes (1,024 in total) placed in the geniculate recipient layer of the primary visual cortex (V1) and in area V4 of the ventral visual stream. Monkeys equipped with such implants were able to recognize simple shapes, motion and letters (Chen et al., 2020).

##### Orion

This system consists of a camera, a computer and a subdural array of 60 surface electrodes applied to the medial occipital lobe. After processing of the video image, the information is transmitted wirelessly to the array. A preliminary study in one blind patient demonstrated the safety and basic functional aspects of the device. Ongoing clinical trials that started in late 2017 have so far included five blind patients with a follow-up planned for 5 years (Niketeghad and Pouratian, 2019). Preliminary results indicated that patients were able to perceive phosphenes (Barry et al., 2020).

##### ICPV Project

The Intracortical Visual Prosthesis Project (ICVP) uses a Wireless Floating Microelectrode Array (WFMA) consisting of 16 parylene-insulated iridium microelectrodes placed on the surface of the visual cortex, an integrated circuit microprocessor and a microcoil with wireless power and activation. A video camera mounted on eyeglasses or a headband connects to the video processor unit that converts images into a pattern that maps to the array of electrodes. The signal is then transmitted to the telemetry controller located on the head *via* the stimulation modules that distribute signals and power wirelessly to each WFMA module. Human clinical trials are ongoing (Troyk, 2017).

##### Gennaris

This setup consists of a camera mounted on eyeglasses to capture the scene and transmit it to a “Pocket Processor” that extracts useful information and then sends it to the tiles (43 intracortical electrodes per tiles) implanted in layer 4 of the primary visual cortex. Signals are broadcast by a wireless transmitter located at the back of the head (Lowery et al., 2015). Safety tests on experimental animals have confirmed the production of phosphenes, and histological examination reveals minimal damage to the cortex after implantation and that long-term stimulation is possible without adverse events (Lowery et al., 2017; Rosenfeld et al., 2020). The first human clinical trials are planned in the coming years.

#### Retinal Implants

Retinal prostheses have been developed as potential treatments for retinal pathologies such as RP and AMD, which are the leading causes of blindness. In these pathologies, the retinal ganglion cell (RGC) layer is relatively unaffected, making such patients good candidates for intraretinal implantation (Santos et al., 1997; Medeiros and Curcio, 2001). Retinal prostheses are classified according to the locus of the electrode array, i.e., epiretinal, subretinal, and suprachoroidal.

##### Argus Retinal Prostheses

The first retinal prosthesis was the Argus^®^ I, which is an epiretinal array of 16 electrodes wirelessly connected to a computer and a camera. Clinical trials with the implant indicated that patients were able to accomplish simple visual detection and discrimination tasks (Yanai et al., 2007). However, the spatial resolution was inherently limited by the number of electrodes and the distance between them (Caspi et al., 2009). To overcome such limitations, the subsequently the Argus^®^ II that boasts an epiretinal array of 60 (6 × 10) platinum electrodes and better spatial resolution of the transmitted signal. Implanted patients were able to discriminate and recognize 2D and 3D objects, identify large high contrast letters (Stronks and Dagnelie, 2014) localize targets (Ahuja et al., 2011) and detect motion (Arsiero et al., 2011; Luo et al., 2014). Moreover, in a simple navigation task, patients were able to follow a high contrast line on the ground and find a door (Humayun et al., 2012).

##### Alpha-IMS

Alpha-IMS is a subretinal implant placed in an area devoid of photoreceptors with the goal to act as a substitute for the missing photoreceptors. It consists of a chip composed of 1,500 photodiodes that detect light, an amplifier circuit and penetrating electrodes. The amplified signal activates the bipolar cells (Stingl et al., 2013). Using this implant, patients were able to perceive and localize a light source, and detect motion. The second-generation of the device, the Retina Implant Alpha AMS is an improved version with 1,600 photodiodes and increased durability, and is now being tested (Edwards et al., 2018).

##### The Bionic Vision Australia

The Bionic Vision Australia (BVA) is a suprachoroidal implant that reduces the surgical risks of causing damage to the retina. Since the implant is far from the targeted retinal cells, patients demonstrated very poor visual acuity (20/8397) with the device (Ayton et al., 2014).

**FIGURE 3:**
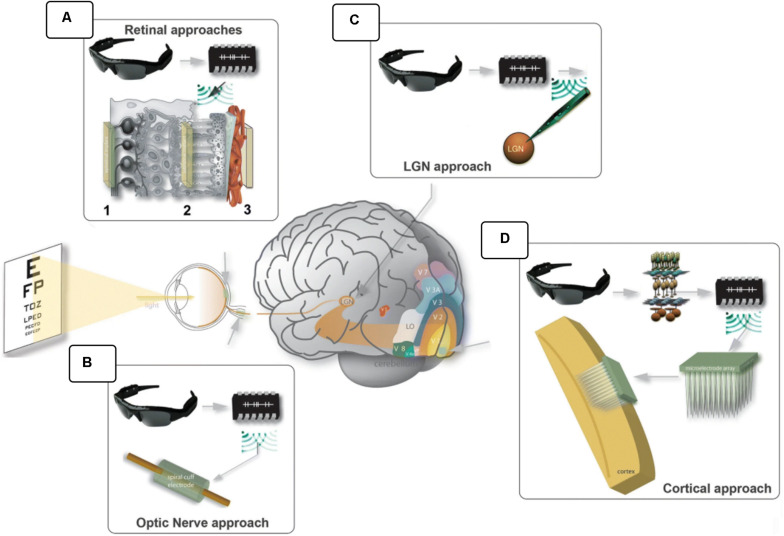
Visual prostheses. In general, the scene is captured by a video camera, processed by a computer unit and sent to the electrical interface that stimulates the visual pathways. Different anatomical locations were explored: **(A)** epiretinal (1), subretinal (2) and suprachoroidal (3); **(B)** the optic nerve; **(C)** the lateral geniculate nucleus; and **(D)** the visual cortex. (modified from Fernandez, 2018; https://creativecommons.org/licenses/by/4.0/).

Figure 3 illustrates four types of invasive implants (retinal, in the optic nerve, thalamic and cortical).

### Non-invasive Brain Interfaces Through Touch and Audition

While invasive interfaces require decisive surgery and have not proven their efficacy, new attempts have been made in developing non-invasive devices. Since the beginning of the 20th century, researchers have developed sensory substitution systems to replace vision with other senses like touch and audition.

#### Electronic Aids for Reading

As described in section “Education Through Touch: From Diderot to Braille and Howe,” Braille brings to blind people a universal writing and reading system. However, the blind people must still rely upon sighted persons to translate printed texts into Braille, or to provide audio transcripts. Several devices have been designed to give the blind broader independence in reading.

The Optophone, developed in 1912, was one of the first sensory aid systems to transduce light into sound. First designed for enabling independent mobility, it later found application as a reading aid. Equipped with the device, which applies mechanical signals to the hand, some blind people were able to read at a rate up to 60 words per minute (d’Albe, 1920). This early success inspired some scientists to consider the incredible potential of the tactile sense for sending “visual” information to the brain. Indeed, Geldard (1957) developed a vibrotactile device based on a communication code like Morse code that could transmit individual letters to the reader (Geldard, 1957). Bliss et al. (1963) took the idea one step further by using air puffs to the chest as tactile stimulators, and found that (with training) blind subjects could perceive apparent motion with good spatial and temporal acuity (Bliss et al., 1963). In 1966, Bliss went on to design a system of vibrotactile stimulators consisting of 96 piezoelectric pins, each connected to photocells, which enabled the blind to perceive printed texts. By placing their index finger on the piezoelectric grid, users could feel the vibrations corresponding spatially to the letters. After 50 h of practice, some participants could read at a speed of 30 words per minute, thus one third of the rate for skilled Braille readers (Linvill and Bliss, 1966). This system became commercially available in the 1970s under the name Optacon (Optical to Tactile Conversion), but did no longer find great success in the 1990s since it was surpassed by the advent of scanners equipped with optical character recognition software that became generally less expensive, easier and faster to use to access printed literature without vision (Stein, 1998; Kendrick, 2005).

#### Electronic Travel Aids to Assist Mobility

To improve personal safety during navigation, electronic travel aids (ETAs) mainly function on the echo principle of active energy-radiating systems. Indeed, most ETAs are devices that detect obstacles by emitting a form of energy and capturing its reflection with a sensor. ETAs can deliver to the blind user information about looming obstacles, communicated by easily understandable auditory or tactile stimulations.

##### Electromagnetic Radiation

Electronic travel aid devices working on the emission of electromagnetic radiation (light), often functioned through optical triangulation (Benjamin, 1974). The light rays reflected by the tangible surface (or obstacle) enter the sensor (photodetector) at various angles depending on the object’s distance. The incident angle thus encodes distance information. The first effective ETAs, known as Obstacle Detectors, enabled the detection of objects by sending a single beam of light from a hand-held flashlight-like source. They signaled the detected obstacle with a vibration of the handle, thus permitting the users to detect and avoid obstacles in various environments (maze, street, store) but users proved to be slower than with their habitual white cane (Benjamin, 1968). Furthermore, these devices could not detect changes in floor texture or elevation and participants, thus, preferred using them in combination with their cane. This finding led to the development of a system combining the cane and the light beam, the laser cane, which was equipped with three laser sources pointing at different angles (downward, forward, and upward), thus aiming to extend the range of the cane while enabling the detection of higher obstacles (Benjamin, 1974). The latest prototype, the laser cane N-2000, was used in the 2000s, but is no longer available because it was significantly more expensive than similar ultrasonic ETAs (Roentgen et al., 2008; Li, 2015).

##### Scanning With Ultrasounds

**FIGURE 4:**
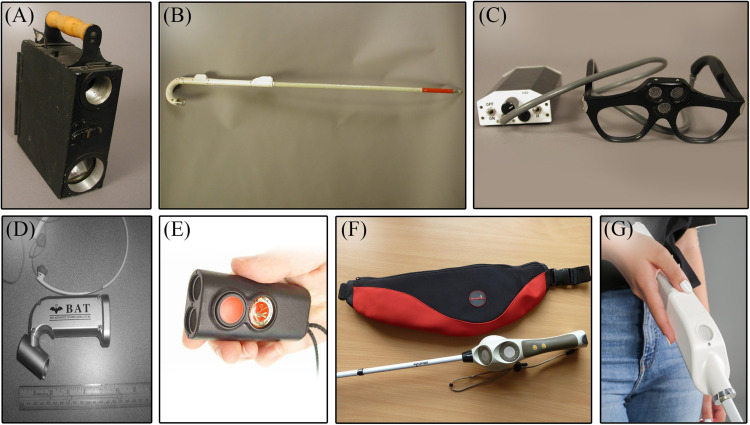
Non-invasive aids. On the upper row are historic ETAs that are no longer available (photo courtesy AER O&M Division Warren Bledsoe Archives, American Printing House for the Blind). **(A)** Signal Corps Obstacle Detector; **(B)** C-4 laser cane; **(C)** Sonic Guide; **(D**) “K”-Sonar [from Penrod et al. (2009); with the permission of W. Penrod]; **(E)** Miniguide (GDP Research); **(F)** UltraCane (from http://www.ultracane.com, with the permission of Sound Foresight Technology Ltd); and **(G)** WeWALK smart cane (from https://wewalk.io/en/, with the permission of WeWALK Tech Co.).

Ultrasonic signals have a slower propagation speed than light, which naturally leads to longer reflection delays, allowing for more precise measurements of distances compared to optical triangulation (National Research Council, 1986). For this reason, contemporary ETAs still use ultrasounds. One of the first successful ultrasonic ETAs was Russell’s Travel Pathsounder, a pendant-like device that emitted a conical ultrasonic beam for obstacle detection. It reduced collision risks by signaling obstacles in the immediate navigational environment with simple sounds and vibrations as warnings (Russell, 1967). A later device, the Sonic Guide (successor to the Sonic Torch and Binaural Sensory Aids), enabled some degree of object discrimination and localization with more complex auditory cues (Kay, 1964). The Sonic Guide technology became the foundation of the “K”-Sonar (Penrod et al., 2009), a smaller compact sensor that can be fixed to the white cane. Ultrasonic ETAs such as the “K”-Sonar (BAT Technologies) and the Miniguide (GDP Research) are still being manufactured (Smith and Penrod, 2010), notably the UltraCane (Sound Foresight Technology), which combines two ultrasonic sensors to a traditional white cane (Hoyle and Waters, 2008), and the newer WeWALK smart cane (WeWALK, 2019), an innovative “all-in-one” primary aid. It combines the traditional white cane with a single ultrasonic sensor, a touch pad and a voice assistant for smart control of the user’s smartphone without requiring the other hand. Examples of ETAs are depicted in Figure 4.

#### Modern Sensory Substitution

Non-invasive sensory substitution endeavors to use a non-visual sensory input to stimulate the visual cortex and other brain areas related to vision, all *via* natural rerouting of existing sensory channels (Foulke et al., 1986). These methods thus exploit the brain’s natural adaptation mechanisms. They offer new possibilities to “restore” visual function in blind people, and have attracted considerable interest since their inception. Paul Bach-y-Rita did the pioneering work on sensory substitution in the 1970s. At a time when most scientists believed the visual areas of the blind to be atrophied and non-functional, Bach-y-Rita argued that the visually deprived brain could readapt, since it had only lost the peripheral systems (i.e., eye, retina). In *Brain Mechanisms in Sensory Substitution*, Bach-y-Rita (1972) recounted that the images captured by the eyes travel to the brain in the form of neuronal signals. Therefore, sight is not mediated by the eyes, but by the brain’s interpretation of incident electrical signals, based on hard-wiring of the brain, but also informed by memory, learning, contextual interpretations, and many other factors (Bach-y-Rita and Aiello, 1996). According to Bach-y-Rita, people living with blindness could regain access to the missing visual input if only were made accessible *via* their intact senses (Bach-y-Rita et al., 1969). With this idea in mind, Bach-y-Rita designed the Tactile Vision Substitution System (TVSS), a sensory substitution system for transmitting visual information through the skin surface of the back. A camera captured visual information that was then transmitted over an electro-tactile grid which activated skin receptors that sent visual information to the brain, where it is processed and perceived. Case study investigations showed us that it is possible, with some learning, to feel and interpret different patterns drawn on the skin of the back and then to use that information to judge distances and even catch moving objects. Several models of the TVSS were manufactured with the goal of greater portability and increased effectiveness in the visual domain. Bach-y-Rita investigated the use of electrodes arrays on the fingers (Kaczmarek et al., 1994), on the abdomen (Kaczmarek et al., 1985) and on the tongue (Bach-y-Rita et al., 1998; Sampaio et al., 2001). He concluded that the tongue was the best option based on several criteria. First, the tactile sensitivity of the tongue is significantly greater than that of the skin of the back or fingers. Second, the cortical surface for the tongue is larger than the corresponding surface for the entire back. Third, the tongue’s tactile receptors are closer to its surface, while the saliva, which is an electrolytic solution, assures electrical contact between the electrodes and the tongue (Bach-y-Rita, 2004). Consequently, the tongue requires significantly less voltage and current than does the fingertip in order to perceive electrotactile stimulations (Bach-y-Rita et al., 1998; Bach-y-Rita and Kaczmarek, 2002).

#### First Generation of SSDs: TDU, vOICe, and PSVA

The Tongue Display Unit (or TDU) transmits visual input to the tongue in the form of electrotactile pulses. It is composed of a 20 × 20 matrix array of small circular electrodes that is placed on the tongue, a laptop computer and a webcam attached to eyeglasses. The visual image is translated into electrotactile pulses and thus “drawn” in real time with the application of electrical currents on the tongue. Several studies have shown that TDU allows the blind to perceive light sources (Nau et al., 2013; Lee et al., 2014), movement (Ptito et al., 2009; Matteau et al., 2010), and to recognize shapes (Ptito and Kupers, 2005; Vincent et al., 2014), objects (Williams et al., 2011; Nau et al., 2015b), and letters (Chebat et al., 2007b; Pamir et al., 2020). Users of the TDU are even able to navigate in an obstacle course (Chebat et al., 2011). Furthermore, the estimated “visual acuity” of the tongue attained an acuity of 1/90 in trained users (Chebat et al., 2007b), which meets the criterion of low vision that is sufficient to perceive environmental shapes (Ptito et al., 2012) and useful for many visual tasks [reviewed in Stronks et al. (2016)].

Since blind people are also able to perform certain spatial tasks using sound cues (Kellogg, 1962; Bassett and Eastmond, 1964), auditory SSDs have been developed to enhance this skill (Meijer, 1992; Capelle et al., 1998; Bronkhorst and Houtgast, 1999; Kay, 2000). The best known auditory-to-vision SSD was described by Meijer (1992), who named their device the vOICe, where the capitals O, I, and C represent the exclamation “Oh, I see!” The system offers “functional vision” by converting images captured by a video camera to different soundscapes. To do so, the algorithm uses a scanning technique that divides the field of view (FOV) into a matrix of pixels. Initially, the system used a 64 by 64 pixels matrix containing 4,096 elements, but has since evolved to generate a much higher resolution of up to 25,344 pixels (Striem-Amit et al., 2012b). The algorithm analyses every column of pixels in a left to right sequence to translate vertical position to the frequency domain and horizontal position to the duration of the sound. As for colors, they are integrated in a scale of 16 shades of gray so the system can convert luminosity to different sound amplitudes (Meijer, 1992). Striem-Amit et al. (2012b) evaluated the audio-visual acuity of the vOICe users after receiving 73 h of training with the device, more than half of whom had attained a visual acuity of 20/320 which outclasses the threshold of blindness (20/400) defined by the World Health organization. Moreover, several studies with the vOICe demonstrated that blind individuals can learn to identify geometric forms and shapes (Amedi et al., 2007), read (Striem-Amit et al., 2012a), locate objects in space (Auvray et al., 2007) and even learn virtual maps (Jicol et al., 2020). Since then, a new version of the vOICe has been developed to add color information to the mixture of the visual information given by the device. Named the Eyemusic, it performs a spectral analysis of the image and links specific colors with recordings of different musical instruments (Abboud et al., 2013, 2014). Therefore, the device simultaneously conveys spatial information and color, thus enhancing the user’s comprehension of space. Furthermore, blind individuals trained with the device were able to recognize facial expressions with the device (Abboud et al., 2013; Arbel-Yaffe and Amedi, 2016).

Another auditory-to-vision SSD known as the prosthesis for substitution of vision with audition (PSVA), has a field of view (FOV) divided in a differential resolution structure, in which the center contains additional pixels for a higher resolution thus mimicking the human retina and its fovea, which mainly serves for pattern recognition while lower resolution in the periphery allows spatial localization and movement detection (Capelle et al., 1998). The PSVA offers a sonification strategy similar to that of the vOICe by assigning each pixel a sinusoidal tone at distinct pitches and modulated by the gray level intensity. However, instead of scanning images, it uses binaural differences and tone intensity to code for horizontal positioning, while different frequencies are used for vertical positioning, thus, exploiting the natural mechanisms of human hearing (Gulick et al., 1989). Few behavioral studies have been done with this device. However, studies in blind individuals have shown that the PSVA imparts efficient pattern recognition (Arno et al., 2001), spatial localization (Collignon et al., 2007), and depth perception (Renier et al., 2005b).

#### New Generation of SSDs: Eyecane, SoV, and GSSD

The newer generation of SSDs is not designed to restore high resolution vision, but rather to gather and transmit specifically chosen cues to provide greater independence to the user in a specific task such as navigation. The Eyecane, for example, is a minimalist SSD that uses a “point-to-distance” technology as an aid to navigate. In brief, the device uses infrared light sensors to detect a single point in front of the user and calculate the distance between the detected obstacle and its sensor. The device then conveys this information in the form of tactile (vibrations) and auditory cues such as, higher the vibrations and sounds, as one approaches the object (Maidenbaum et al., 2014b). With its small and handy structure, it is designed to bring greater freedom than is afforded by the white cane while also providing superior detection range (Buchs et al., 2014, 2017; Maidenbaum et al., 2014c). This device enables quick and efficient perception of the distance between the user and obstacles in the environment by using sweeping motions, analogous to those with the white cane, thus requiring minimal additional training. Using this device, CB participants were able to navigate in a Hebb–Williams maze as efficiently as sighted participants (Chebat et al., 2015), and were able to transfer spatial information from a virtual environment to the real world (Chebat et al., 2017).

Another promising navigational aid called the Sound of Vision (or SoV) was recently developed. The SoV uses a combination of sensors and a video camera (both mounted on the forehead) to convey the 3D information of the environment, namely depth, positioning, form, and size, *via* a hybrid audio-haptic signal. The haptic signal is delivered on the skin of the abdomen to inform the user of the closest obstacle (Caraiman et al., 2017). As for the auditory signal, the system divides its FOV into a 3 by 5 matrix, in which every sector of the matrix codes and translates depth and direction information into spatialized “popping bubbles” sounds. Thereby, the user can extract the form and the position of an obstacle, while estimating its distance (Hoffmann et al., 2018). Interestingly, the SoV system simplifies its signal by encoding only the closest obstacles in the user’s path thus reducing the cognitive demand placed on the user (Caraiman et al., 2017).

**FIGURE 5:**
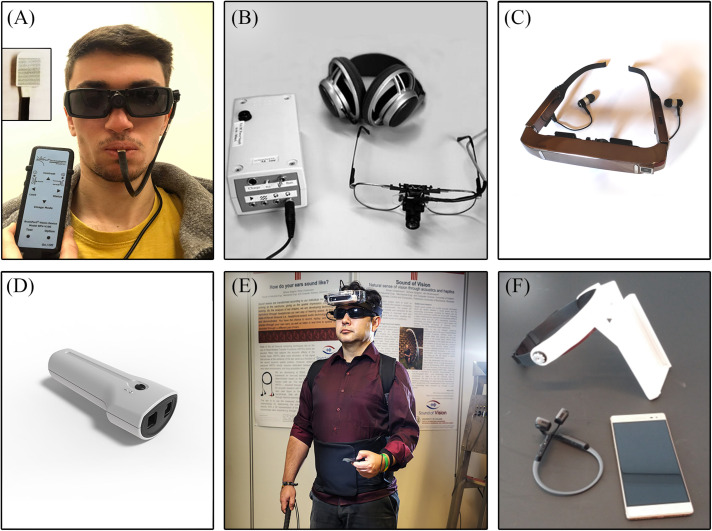
Illustrations of SSDs. **(A)** Tongue Display Unit (TDU); **(B**) vOICe; **(C)** Prosthesis for Substitution of Vision with Audition (PSVA) [from Collignon et al. (2007); with the permission of O. Collignon]; **(D)** Eyecane; **(E)** Sound of Vision (SoV) [adapted from Hoffmann et al. (2018)]; and **(F)** Guidance Sensory Substitution Device (GSSD).

Since smartphones are increasing in popularity in the blind community (Kacorri et al., 2017), SSDs have come to exploit their accessibility and simplicity by making available useful applications. Once such novel smartphone application called the Guidance-Sensory-Substitution-Device (or GSSD) guides users through obstacles, thus increasing their navigational independence. The GSSD uses the cameras of smartphones to capture the environment and bone-conducting earphones to inform the individual of oncoming obstacles by broadcasting horizontally spatialized sounds. The GSSD conveys a simple auditory output based on the point-to-distance principle, while signaling every potential obstacle with a singular sound source that depicts the distance of closest edges from the user. By this means, the user can associate each sound source to a specific obstacle and then plan her/his route through space (Paré et al., 2019). Illustrations of the SSDs are shown in Figure 5.

## Sensory Substitution and Cross-Modal Rewiring of the Brain in Congenital and Late Blindness

### Sensory Substitution

Studies on sensory substitution in CB concur in showing their superior spatio-cognitive skills, which again show that the blind have come to possess certain supernormal skills for sound localization (Lessard et al., 1998) and proprioception (Loomis et al., 1993). In addition to spatial tasks, several other studies also show a marked perceptual advantage for performing cognitive tasks (Muchnik et al., 1991; Röder et al., 1999; Bavelier and Neville, 2002; see also Kupers and Ptito, 2014), verbal memory (Amedi et al., 2003), and attention (Muchnik et al., 1991; Röder et al., 1996, 1999; Liotti et al., 1998). Since the pioneering work demonstrating that the visual cortex of CB can, with training, be recruited by tactile stimulation, i.e., training-induced plasticity (Sadato et al., 1996; Ptito et al., 2005), the bulk of subsequent studies has confirmed the activation of the visual cortex in tactile, auditory, and olfactory tasks [reviewed in Kupers and Ptito (2014) and Nau et al. (2015a)]. Interestingly enough, not only is the visual cortex activated by tactile stimuli but the tactile motion and shape information are funneled into the dorsal (Ptito et al., 2009) and ventral visual pathways (Ptito et al., 2012), respectively. This phenomenon has also been shown upon auditory stimulation of encoded visual information (Collignon et al., 2007; Striem-Amit and Amedi, 2014; Arbel-Yaffe and Amedi, 2016). This recruitment of visual areas for tactile and auditory tasks gives CBs an advantage for the use of sensory substitution devices (Ptito et al., 2005), and allows them to significantly increase their performance after only a few hours of training (Sampaio et al., 2001). Moreover, the brain areas activated when exploring a virtual maze using a tactile-to-vision substitution device roughly matched the areas activated when sighted people explored a virtual maze using vision, but differed from those activated in blindfolded sighted controls. We have previously shown that the occipital cortex and the hippocampal/parahippocampal complex are involved in route recognition in CBs, similar to sighted people performing the same tasks with opened eyes (Kupers et al., 2010; Chebat et al., 2020). This network of brain regions is important for navigational behavior in sighted people (Maguire et al., 1998, 2000; Schindler et al., 2004; Epstein, 2008; Browning et al., 2009; Squire, 2009). These natural mechanisms of adaptation in the blind brain should be used to guide the development of training programs using SSDs, since they highlight the inherent ability of the brain to recruit task-specific areas when using substituted sense modalities (Chebat et al., 2018b).

### Cross-Modal Plasticity

**FIGURE 6:**
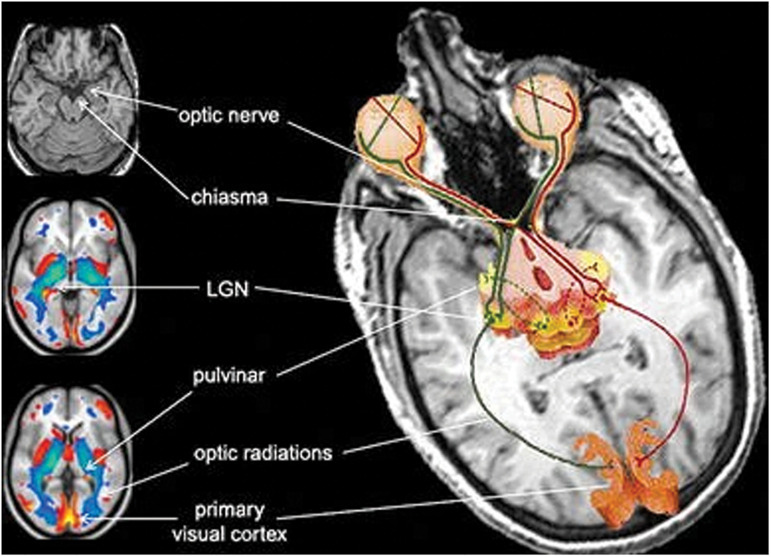
Atrophy of the components of the visual system in congenitally blind individuals [from Ptito et al. (2008); with the permission of Springer Nature, license # 4986491335589].

Since congenital blindness and early onset of vision loss alters the retinofugal projections to the visual cortex, the blind brain undergoes a massive anatomical reorganization leading to cross-modal plastic reconfigurations of sensory pathways. This is possible because the brain has a natural ability, called neuroplasticity, to adapt itself in response to every perturbation in the external and the internal environment. The first structural/functional studies on the visual system of blind people using magnetic resonance imaging (MRI) and positron emission tomography (PET) found significant alterations not only in the white matter tracts including the optic nerves, the optic chiasm and the optic tracts (Breitenseher et al., 1998; Ptito et al., 2008) but also relative reductions of the gray matter volume in the visual thalamus (the lateral geniculate nucleus, and posterior pulvinar), and striate and extra-striate visual cortices (Shimony et al., 2006; Ptito et al., 2008; Cecchetti et al., 2016). Other volumetric reductions were reported in the brain commissural systems such as the splenium of corpus callosum (Ptito et al., 2008; Tomaiuolo et al., 2014; Cavaliere et al., 2020), accompanied by an enlargement of the anterior commissure (Cavaliere et al., 2020). In addition, regions connected to the dorsal visual stream such as the hippocampus were also reduced in volume (Chebat et al., 2007a; Fortin et al., 2008). Cortical thickness is increased in the primary visual cortex of the congenitally blind (Jiang et al., 2009; Kupers et al., 2011) accompanied by a supra-metabolic activity therein (De Volder et al., 1997; Kupers and Ptito, 2014). Figure 6 shows the atrophy in various components of the visual system of CB individuals.

**FIGURE 7:**
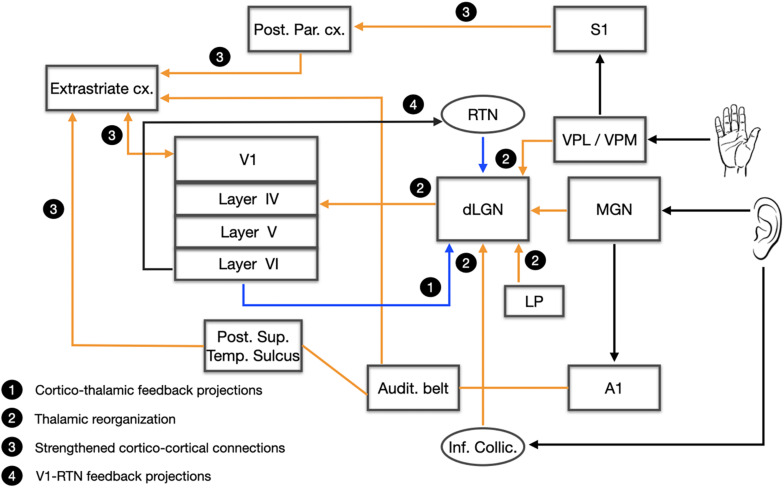
The rewired blind brain. A schematic representation of the reorganization of the blind brain. Four networks are presented: **(1)** Cortico-thalamic feedback projections; **(2)** Thalamic reorganization; **(3)** Strengthened cortico-cortical connections; **(4)** V1-RTN feedback projections. Audit. Belt, auditory belt; A1, primary auditory cortex; Cx, cortex; dLGN, dorsal lateral geniculate nucleus; LP, lateral pulvinar; MGN, medial geniculate nucleus; Post. Par. Cx.; posterior parietal cortex; Post. Sup. Temp. Sulcus, posterior superior temporal sulcus; RTN, reticular thalamic nucleus; S1, primary somatosensory cortex; VPL, ventral posterolateral nucleus; VPM, ventral posteromedian nucleus; V1, primary visual cortex.

Furthermore, magnetoencephalography has provided evidence for increased functional connectivity of the occipital cortex with auditory and somatosensory areas (Ioannides et al., 2013; Kupers and Ptito, 2014; Müller et al., 2019), as likewise shown in studies using transcranial magnetic stimulation (Wittenberg et al., 2004; Kupers et al., 2006). Other functional connectivity studies revealed stronger connections of the visual cortex with somatosensory (Shu et al., 2009), auditory (Watkins et al., 2013; Burton et al., 2014), and language areas (Bedny et al., 2011; Butt et al., 2013). Finally, a recent resting state functional magnetic resonance imaging (rsfMRI) study (Heine et al., 2015) revealed increased functional connectivity within both the ventral and the dorsal visual streams in congenitally blind participants along with a stronger functional connectivity between the occipital cortex and language areas, and regions involved in tactile (Braille) processing such as the inferior frontal gyrus (Broca’s area), the thalamus, the supramarginal gyrus and the cerebellum (Heine et al., 2015). Taken together, most anatomical studies concur in showing that the tactile or auditory information reach the visual cortex of the blind through both a multi-synaptic cortico-cortical pathway (Ptito and Kupers, 2005) and also through a direct thalamo-cortical pathway (Kupers and Ptito, 2014; Murphy et al., 2016; Müller et al., 2019). The cross-modal rewiring of the blind brain is illustrated in Figure 7.

### Late Blindness

The study of late acquired blindness (LB) poses a completely different challenge than early acquired blindness (Chebat et al., 2018b). LB subjects have a visual system that has developed normally until vision loss and basically, they possess a visual brain similar to that of seeing people. Two important parameters were and still are often neglected in studies on late blindness, namely the onset and duration of blindness, which led to the contradictory results reported in the literature. As of now, most of the studies on sensory substitution only tested CB individuals or a mix of LB subjects without considering onset and duration of blindness. It is known that the neuroplastic processes that accompany the onset of blindness are less strong in LB, taking into account that plasticity is highly dependent on critical periods of development (Sadato et al., 2002; Noppeney, 2007; Jiang et al., 2009). One could therefore argue that once this critical period is over, the brain is less likely to adapt itself to a new condition. Nonetheless, a number of studies have reported neuroanatomical differences between CB and LB, and LB and subjects with normal vision, which challenges the rigidity of critical periods in the brain (Heimler and Amedi, 2020). For example, cross-modal plastic processes have been usually found in CB whose visual cortex is activated by other senses like audition, touch and even smell [reviewed in Kupers and Ptito (2014)]. These plastic manifestations are also found in LB but in the extra-striate visual areas (Sadato et al., 2004; Renier et al., 2005a; Amedi et al., 2007; Collignon et al., 2013) and in the splenium of the corpus callosum (Shi et al., 2015; Cavaliere et al., 2020).

Moreover, only a handful of studies have been devoted to the perceptual, cognitive and navigational abilities of late blind individuals (Chebat et al., 2018b). Differences were shown mainly in auditory capacities and navigational strategies when compared to CB [reviewed in Kupers and Ptito (2014)]. For instance, LB have inferior abilities than CB in using binaural and monaural cues for localizing sound sources (Voss et al., 2004) and in echolocation (Dufour et al., 2005) but have better performances in auditory spatial bisection (Amadeo et al., 2019). Moreover, before vision loss, subjects learn to navigate using mostly allocentric strategies. Without vision, LB has to adapt their strategies by transiting into egocentric point of views with only tactile and auditory cues like CB individuals do. Although LB can learn to use SSDs very efficiently (Lee et al., 2014; Chebat et al., 2015, 2017; Paré et al., 2019), it is clear that they do not possess the same skills as CB (Wan et al., 2010; Chebat et al., 2015, 2017). This is probably due to the fact that the cross-modal changes witnessed in the late blind are limited compared to that of CB (Park et al., 2009; Reislev et al., 2017; Wen et al., 2018). Therefore, the visual experience of LB seems to impair their ability to use SSDs compared to CB and their visual experience seems to be detrimental to cross-modal rewiring of the brain. Invasive devices however, are geared specifically toward LB since their technology requires visual experience (Castaldi et al., 2016).

## Discussion

### Brain-Machine Interfaces to Assist the Blind

In this chapter, we briefly described the history of blindness from ancient to modern times. We then addressed the various means that have been used to help blind individuals throughout history, with an emphasis on modern technologies. We divided these aids into two categories: invasive prostheses and non-invasive brain interfaces.

#### Invasive Techniques and Their Limitations

**FIGURE 8:**
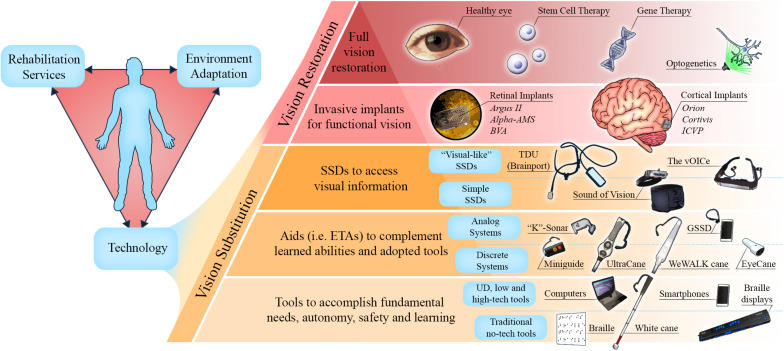
Technologies to assist the blind. (Left) Blind individuals can gain autonomy *via* a combination of the following fundamental and interlinked factors: rehabilitation services, environment adaptation, and technology. (Right) A model of present and future technologies and therapies to substitute and restore vision. At the bottom of the pyramid are simple devices, or tools, that are currently adopted by blind individuals to meet their fundamental needs. At each level, technologies aim to enable more tasks toward the goal of full vision substitution or full vision restoration. However, due to limitations discussed in this review, the higher the technology or therapy on the pyramid, the greater the obstacles to its application and adoption by blind individuals. ETAs, electronic travel aids; SSDs, sensory substitution devices; UD, universal design.

The retina and the visual cortex have been the site of choice for most of the visual prostheses employing electrical stimulation. Located at both extremes of the visual pathways, they are more surgically accessible than are deep brain structures such as the optic nerve and the LGN. Targeting these terminal sites presents certain advantages and challenges. In general, the electrical stimulation of the visual pathways induces phosphenes. In epiretinal prosthesis, the evoked phosphenes have proven to be highly variable and dependent on the activation of passing axon fibers by the implanted electrodes (Beyeler et al., 2019). Moreover, the retina undertakes complex processing of visual inputs, extending from the spatiotemporal integration of light by the photoreceptors to the output of RGCs to the deep visual relay centers (Demb and Singer, 2015). Therefore, stimulation strategies should take into account the structural and functional properties of the retina in order to reproduce a naturalistic activity in the RGC layer for downstream processing in cortical visual areas (Nirenberg and Pandarinath, 2012). The visual cortex is the primary recipient of the retino-geniculate input, which is then processed further in higher order visual areas. However, the neuronal and processing complexity is much higher therein, making it difficult to obtain a meaningful perception through electrical stimulation of the retina only. A major limitation of this approach is that retinal neurons activation affects the activation/inhibition balance that influences the signal propagation to higher order cortical areas (Bosking et al., 2017). While the visual cortex was the first site of stimulation to be explored, it took longer time to reach the safety standards required for human clinical trials, given the obviously more invasive surgical procedures involved. This is why most of the clinical trials have hitherto employed retinal prostheses that lead to letter and object recognition and navigation. Moreover, the best visual acuity offered to date by visual prostheses still falls below the threshold of visual acuity that defines blindness (20/400). For the present, the surgical risks remain too great to justify the few benefits provided by invasive prostheses. Indeed, major neurosurgical procedures are inherently dangerous and can cause deleterious complications such as infection, inflammation, and neurodegeneration along with other neurological problems. Another element restricting the use of these technologies is that they are not appropriate for people who were deprived of vision since birth. Their efficacy relies on the presence of a normally developed visual system with a visual repertoire acquired through experience (Reich et al., 2012). In CB, who were deprived of visual inputs since birth, the visual system undergoes cross-modal rewiring that leads to a massive reorganization of non-visual inputs to the visual cortex (see Figure 8) [reviewed in Kupers and Ptito (2014)] which disfavors the use of surgical prostheses.

Although electrical stimulation has been extensively used in experimental setting, other stimulation strategies are under investigation. The new technique of optogenetics uses viral vectors to genetically modify cells to express rhodopsin, enabling the modulation of neuronal population activity by light with high spatiotemporal resolution. This technique has been explored both for the retina and the visual cortex. Current clinical trials are testing the feasibility of using optogenetics to render the RP patients sensitive to light (Farnum and Pelled, 2020).

#### Non-invasive Devices

Sensory substitution and electronic aids have an advantage over invasive technologies by virtue of exploiting the plasticity mechanisms that naturally operate in the blind brain when trained in other modalities. While some SSDs provide the blind a “visual” perception that exceeds the World Health Organization legal blindness threshold and with no health risks, several factors limit their use outside laboratories. For instance, the spatial resolutions of available devices are limited by the targeted sensory modality. Indeed, since hearing and touch both have lesser spatial bandwidth than natural vision (Bach-y-Rita et al., 1969; Bach-y-Rita, 1972; Apkarian-Stielau and Loomis, 1975; Wiley et al., 1986; Ashmead et al., 1990), a direct translation of visual information to either touch or hearing inevitably results in loss of details (Loomis et al., 2012). Moreover, SSDs are generally designed to assist the blind without consideration of their opinions, contribution and cooperation, and have only been validated in heterogeneous populations of late onset and congenitally blind individuals. This obviously impacts the results on behavior [reviewed in Kupers and Ptito (2014) and Chebat et al. (2018a)]. Important factors that could influence the way SSDs are used and appreciated by users have hitherto been underestimated (Elli et al., 2014). As a consequence, numerous devices have proven to be either too complex or too expensive to operate in real life situations. Indeed, many devices require several hours if not days and months of training that discourage the blind for using them (i.e., the vOICe). This is a major impediment to their broader implementation since most of the attentional resources of the users are focused on decoding the SSD signal instead of understanding their surroundings. This attentional misplacement leads to cognitive overload and exhaustion in complex environments (Elli et al., 2014; Pissaloux and Velázquez, 2018). Indeed, Consequently, the blind community in general is not highly motivated to adopt these apparatuses (Elli et al., 2014; Maidenbaum et al., 2014a; Chebat et al., 2018a).

A more compelling solution for individuals living with blindness is presented by the new minimalist SSDs (Eyecane, GSSD) and ETAs (Miniguide, UltraCane, and WeWALK cane), which are the mainstays of assistive mobility technologies currently used and introduced in O&M training (Smith and Penrod, 2010). Their broader application is favored by the greater simplicity of their signals and ease of use, which makes them acceptable supplementation aids. Furthermore, the advent of computers and smartphones with accessible software (built with universal design) allows more flexibility and opportunities for individuals to share their experiences with the rest of the population. As an example, screen-reading software, optical character recognition software, and travel related applications adapted for the blind can all be accessed through smartphones, and have become increasingly popular amongst individuals with blindness (Kacorri et al., 2017). As in the case of the GSSD (Paré et al., 2019), sensory substitution could also benefit from the processing capacities of smartphones by being designed as downloadable smartphone applications. There is also scope for adapting the urban environment better to suit the needs of individuals living with disabilities, and to increase their safety and autonomy as stated in *American Disability Act* (ADA, 1990). Indeed, the increasing number of measures such as the installation of tactile plates and auditory pedestrian signals are good examples of such universal design. This calls for the promotion of widespread standardization of such enabling measures, and also calls for further research and development of technologies, like presenting 3D printed tactile maps in buildings and in public places. Moreover, artificial intelligence is a promising venue as it can provide blind individuals with devices or applications equipped with image recognition software for text, faces, objects, and even larger scale environments to enable more efficient interactions and autonomous mobility (Morrison et al., 2017; Kelley, 2018; Zhao et al., 2018). Our view is illustrated in Figure 8, which highlights the present and future methodologies extending from simple vision substitution to full vision restoration through highly sophisticated interventions such as gene therapy, stem cell technology or optogenetics.

## Ethics Statement

Written, informed consent was obtained from the participants for the publication of any identifiable data and images.

## Author Contributions

MP, D-RC, and FS planned the review. MP, D-RC, FS, SP, MB, and ID wrote the manuscript. All authors have contributed equally to this work.

## Conflict of Interest

The authors declare that the research was conducted in the absence of any commercial or financial relationships that could be construed as a potential conflict of interest.
